# Supplementary material for: Modeling glioblastoma heterogeneity as a dynamic network of cell states
Source: Mol Syst Biol. 2021 Sep 16;17(9):e10105. doi: 10.15252/msb.202010105 (PMC8444284; doi:10.15252/msb.202010105)
Supplement: Supplementary file 5 — Source Data for Figure 3 [file MSB-17-e10105-s001.zip › Figure3A_sourcedata/GSEA_3065/hallmarks_state3.GseaPreranked.1623416274744/HALLMARK_INFLAMMATORY_RESPONSE.html]

Details for gene set HALLMARK\_INFLAMMATORY\_RESPONSE[GSEA]

|  || Dataset | state3 |
| Phenotype | NoPhenotypeAvailable |
| Upregulated in class | na\_pos |
| GeneSet | HALLMARK\_INFLAMMATORY\_RESPONSE |
| Enrichment Score (ES) | 0.4091838 |
| Normalized Enrichment Score (NES) | 1.4875181 |
| Nominal p-value | 0.016483517 |
| FDR q-value | 0.067712545 |
| FWER p-Value | 0.232 |
Table: GSEA Results Summary

  

Fig 1: Enrichment plot: HALLMARK\_INFLAMMATORY\_RESPONSE      
 Profile of the Running ES Score & Positions of GeneSet Members on the Rank Ordered List

  

| PROBE | GENE SYMBOL | GENE\_TITLE | RANK IN GENE LIST | RANK METRIC SCORE | RUNNING ES | CORE ENRICHMENT || 1 | TIMP1 |  |  | 35 | 0.470 | 0.0602 | Yes |
| 2 | EMP3 |  |  | 91 | 0.354 | 0.1022 | Yes |
| 3 | TNFAIP6 |  |  | 158 | 0.307 | 0.1366 | Yes |
| 4 | SELENOS |  |  | 213 | 0.276 | 0.1681 | Yes |
| 5 | DCBLD2 |  |  | 243 | 0.265 | 0.2011 | Yes |
| 6 | ATP2B1 |  |  | 271 | 0.253 | 0.2326 | Yes |
| 7 | ITGB8 |  |  | 463 | 0.206 | 0.2388 | Yes |
| 8 | LDLR |  |  | 498 | 0.199 | 0.2621 | Yes |
| 9 | SLC4A4 |  |  | 555 | 0.192 | 0.2818 | Yes |
| 10 | HAS2 |  |  | 603 | 0.186 | 0.3018 | Yes |
| 11 | ATP2C1 |  |  | 671 | 0.175 | 0.3181 | Yes |
| 12 | P2RX7 |  |  | 723 | 0.167 | 0.3351 | Yes |
| 13 | KIF1B |  |  | 767 | 0.161 | 0.3522 | Yes |
| 14 | ATP2A2 |  |  | 792 | 0.158 | 0.3711 | Yes |
| 15 | OSMR |  |  | 905 | 0.148 | 0.3784 | Yes |
| 16 | TAPBP |  |  | 1055 | 0.134 | 0.3796 | Yes |
| 17 | CX3CL1 |  |  | 1077 | 0.133 | 0.3953 | Yes |
| 18 | IFNAR1 |  |  | 1222 | 0.120 | 0.3951 | Yes |
| 19 | PTPRE |  |  | 1396 | 0.109 | 0.3900 | Yes |
| 20 | GNAI3 |  |  | 1417 | 0.107 | 0.4024 | Yes |
| 21 | PDPN |  |  | 1483 | 0.104 | 0.4092 | Yes |
| 22 | GABBR1 |  |  | 1892 | 0.081 | 0.3733 | No |
| 23 | CD82 |  |  | 1930 | 0.079 | 0.3799 | No |
| 24 | SLC7A1 |  |  | 1936 | 0.079 | 0.3901 | No |
| 25 | SLC11A2 |  |  | 1941 | 0.079 | 0.4004 | No |
| 26 | AXL |  |  | 2020 | 0.075 | 0.4017 | No |
| 27 | ABCA1 |  |  | 2275 | 0.065 | 0.3814 | No |
| 28 | CALCRL |  |  | 2315 | 0.064 | 0.3856 | No |
| 29 | MYC |  |  | 2324 | 0.064 | 0.3934 | No |
| 30 | BST2 |  |  | 2410 | 0.060 | 0.3919 | No |
| 31 | PDE4B |  |  | 2443 | 0.059 | 0.3962 | No |
| 32 | SLC31A1 |  |  | 2466 | 0.058 | 0.4016 | No |
| 33 | MET |  |  | 2522 | 0.056 | 0.4030 | No |
| 34 | NAMPT |  |  | 2583 | 0.054 | 0.4035 | No |
| 35 | HIF1A |  |  | 2661 | 0.051 | 0.4016 | No |
| 36 | ITGA5 |  |  | 2747 | 0.049 | 0.3985 | No |
| 37 | PSEN1 |  |  | 2814 | 0.046 | 0.3972 | No |
| 38 | KCNJ2 |  |  | 2991 | 0.041 | 0.3826 | No |
| 39 | SERPINE1 |  |  | 3090 | 0.039 | 0.3766 | No |
| 40 | CSF1 |  |  | 3363 | 0.031 | 0.3496 | No |
| 41 | IRF1 |  |  | 3457 | 0.029 | 0.3429 | No |
| 42 | IRF7 |  |  | 3530 | 0.027 | 0.3383 | No |
| 43 | ACVR2A |  |  | 3557 | 0.027 | 0.3390 | No |
| 44 | HRH1 |  |  | 3626 | 0.025 | 0.3345 | No |
| 45 | AHR |  |  | 4020 | 0.015 | 0.2913 | No |
| 46 | BTG2 |  |  | 4070 | 0.013 | 0.2874 | No |
| 47 | PLAUR |  |  | 4075 | 0.013 | 0.2888 | No |
| 48 | NFKB1 |  |  | 4528 | 0.001 | 0.2368 | No |
| 49 | LY6E |  |  | 4707 | -0.004 | 0.2168 | No |
| 50 | TPBG |  |  | 5003 | -0.011 | 0.1844 | No |
| 51 | ADORA2B |  |  | 5176 | -0.015 | 0.1666 | No |
| 52 | RAF1 |  |  | 5375 | -0.020 | 0.1466 | No |
| 53 | NMI |  |  | 5592 | -0.027 | 0.1254 | No |
| 54 | KLF6 |  |  | 5627 | -0.028 | 0.1254 | No |
| 55 | NFKBIA |  |  | 5684 | -0.029 | 0.1229 | No |
| 56 | ABI1 |  |  | 5708 | -0.030 | 0.1244 | No |
| 57 | SCN1B |  |  | 5911 | -0.036 | 0.1061 | No |
| 58 | RELA |  |  | 6181 | -0.046 | 0.0813 | No |
| 59 | EIF2AK2 |  |  | 6220 | -0.047 | 0.0834 | No |
| 60 | NDP |  |  | 6256 | -0.049 | 0.0860 | No |
| 61 | RIPK2 |  |  | 6615 | -0.062 | 0.0532 | No |
| 62 | CHST2 |  |  | 7170 | -0.092 | 0.0020 | No |
| 63 | RHOG |  |  | 7526 | -0.119 | -0.0226 | No |
| 64 | RGS16 |  |  | 7541 | -0.122 | -0.0075 | No |
| 65 | IFNGR2 |  |  | 7647 | -0.132 | -0.0016 | No |
| 66 | ADRM1 |  |  | 8001 | -0.186 | -0.0168 | No |
| 67 | SRI |  |  | 8395 | -0.292 | -0.0221 | No |
| 68 | CDKN1A |  |  | 8657 | -0.461 | 0.0108 | No |
Table: GSEA details [plain text format]

  

Fig 2: HALLMARK\_INFLAMMATORY\_RESPONSE: Random ES distribution      
 Gene set null distribution of ES for **HALLMARK\_INFLAMMATORY\_RESPONSE**
